# Monte Carlo Calculations Supporting Patient Plan Verification in Proton Therapy

**DOI:** 10.3389/fonc.2016.00062

**Published:** 2016-03-18

**Authors:** Thiago V. M. Lima, Manjit Dosanjh, Alfredo Ferrari, Silvia Molineli, Mario Ciocca, Andrea Mairani

**Affiliations:** ^1^European Organization for Nuclear Research (CERN), Geneva, Switzerland; ^2^Division of Surgery and Interventional Science, University College London, London, UK; ^3^Fachstelle Strahlenschutz, Kantonsspital Aarau AG, Aarau, Switzerland; ^4^Department of Medical Physics, Fondazione CNAO, Pavia, Italy

**Keywords:** Monte Carlo calculations, treatment plan verification, proton therapy, delta ray effect, dose disturbance

## Abstract

Patient’s treatment plan verification covers substantial amount of the quality assurance (QA) resources; this is especially true for Intensity-Modulated Proton Therapy (IMPT). The use of Monte Carlo (MC) simulations in supporting QA has been widely discussed, and several methods have been proposed. In this paper, we studied an alternative approach from the one being currently applied clinically at Centro Nazionale di Adroterapia Oncologica (CNAO). We reanalyzed the previously published data (Molinelli et al. (1)), where 9 patient plans were investigated in which the warning QA threshold of 3% mean dose deviation was crossed. The possibility that these differences between measurement and calculated dose were related to dose modeling (Treatment Planning Systems (TPS) vs. MC), limitations on dose delivery system, or detectors mispositioning was originally explored, but other factors, such as the geometric description of the detectors, were not ruled out. For the purpose of this work, we compared ionization chambers’ measurements with different MC simulation results. It was also studied that some physical effects were introduced by this new approach, for example, inter-detector interference and the delta ray thresholds. The simulations accounting for a detailed geometry typically are superior (statistical difference – *p*-value around 0.01) to most of the MC simulations used at CNAO (only inferior to the shift approach used). No real improvement was observed in reducing the current delta ray threshold used (100 keV), and no significant interference between ion chambers in the phantom were detected (*p*-value 0.81). In conclusion, it was observed that the detailed geometrical description improves the agreement between measurement and MC calculations in some cases. But in other cases, position uncertainty represents the dominant uncertainty. The inter-chamber disturbance was not detected for the therapeutic protons energies, and the results from the current delta threshold are acceptable for MC simulations in IMPT.

## 1 Introduction

Delivering an appropriate radiation therapy dose starts by preparing the most suitable treatment plan for each patient. This is done by conforming the delivered dose to the clinical target volume and avoiding critical organs (2) in order to limit the observed side effects on the surrounding tissue in the patient. Proton beams, with their defined range, can play an important part in increasing this conformity (3). Monte Carlo (MC) simulations are one of the proposed three different dose calculation algorithms, alongside ray trace and pencil beam. Although MC has been considered the gold standard between these approaches in respect to its accuracy, pencil beam model is mostly used in the treatment plan system (TPS) due to its compromise between accuracy and computational time.

After finding the best solution for how to deliver the dose, a verification process is needed in order to check if the equipment is able to deliver the planned treatment fields. Several methods have been proposed, such as the ones by PSI (4) and MD Anderson (5), but at Italian National Center for Oncological Hadron Therapy (CNAO), the method developed by GSI and used at HIT (1, 6), is adopted. CNAO is a hospital-based hadrontherapy facility equipped with a custom synchrotron and Dose Delivery System (DDS) to provide actively scanned proton beams with energies of 62–227 MeV/u and carbon with 115–400 MeV/u, corresponding to ranges in water of 3–32 and 3–27 cm for protons and carbon ions, respectively (7).

Individual treatment plan verifications in the experimental environment can be very time and manpower intensive, and it is prone to dose delivery uncertainties and setup errors. Molinelli et al. (1) presented CNAO’s quality assurance results for all the patients treatment plans verification that have been performed in CNAO with proton beams concerning 1 year (September 2011–August 2012). Nine cases have been found where the quality assurance warning threshold was exceeded, which is fixed at 3% mean absolute deviation between measurements and TPS. Originally, the possibility explored was that these differences between measurement and calculated dose were related to dose modeling (TPS vs. MC), limitations on DDS, or detectors mispositioned (shift), but other factors were not ruled out, such as oversimplification of the dose modeling.

FLUKA (8, 9) was the MC code chosen for this work due to its demonstrated capabilities (10, 11) and available powerful graphical interface (12).

**Figure 1:**
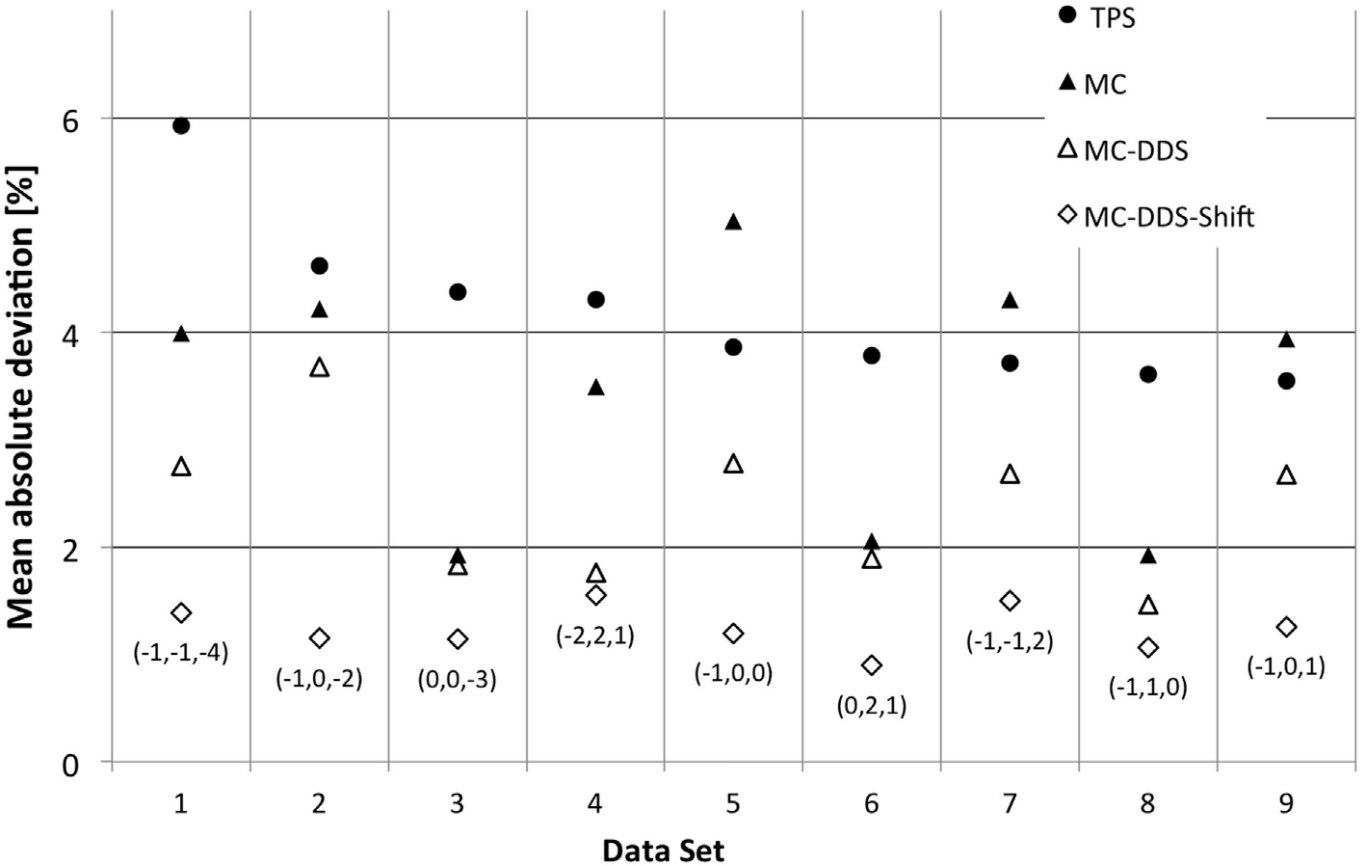
**CNAO patient plan verification results for the nine cases above the warning threshold** (6). For each data set exceeding the warning level, the mean absolute deviation is plotted. Measurements are compared to four different scenarios of calculated values: ● – TPS calculated dose, ▲ – MC simulated dose based on treatment plan data, Δ – MC simulated dose based on DDS log files, and ◊ – MC simulated dose based on DDS log files and corrected for the optimal 3D holder shift. For the last case, the applied translation vector, expressed in millimeters, is also reported between brackets.

In this work, we have evaluated if improvements could be applied to the MC simulations in order to get better agreement with the measured data on these previously described cases (Figure 1). More specifically, we studied the use of more detailed representation of the detectors (13, 14) and the effect of physical processes that these could introduce in the MC simulation results, for example, the required threshold settings for specific scenarios (15).

## 2 Materials and Methods

### 2.1 TPS Patient Plan Verification

The TPS used in CNAO is the CE-marked *syngo*^®^ RT Planning by Siemens AG Healthcare (Erlangen, Germany) version VB10, which is based on TRiP98 (16, 17).

**Figure 2:**
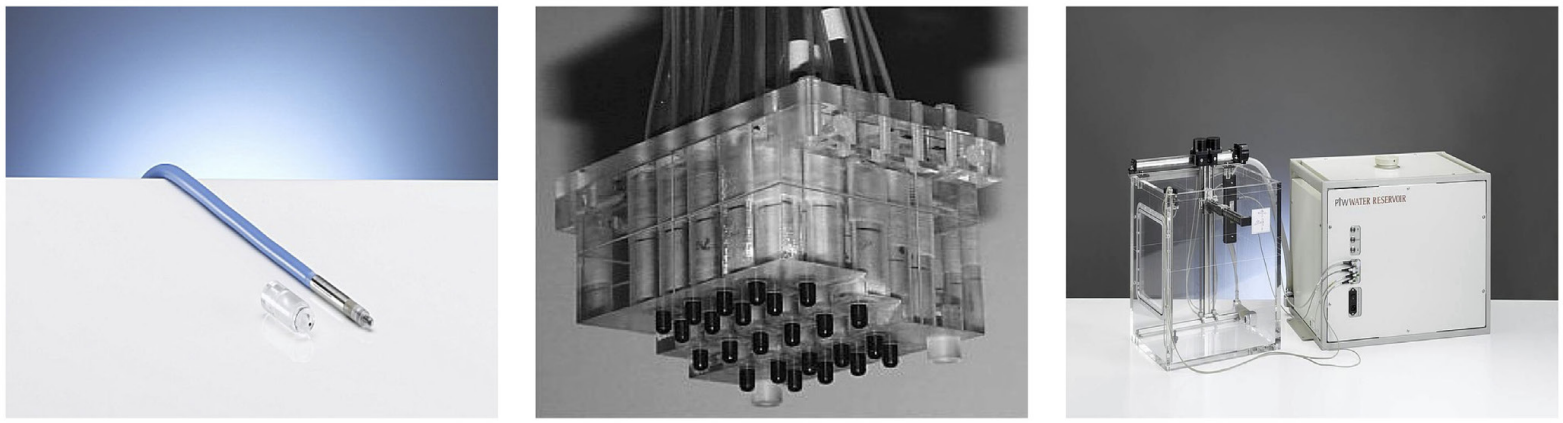
**Different tools used in the treatment plan verification**. The pin point ionization chamber (left), the 3D detector block (center), and the water tank with motorized arm (right) by PTW.

The current CNAO quality assurance procedure (1, 18) specifies that for each patient, plan verification will be performed (6). For this, a water tank with a 3D detector block controlled by a motorized arm (PTW) is used. This enables to measure the deposited dose at different known depths and positions. This detector block provides a support holder for the ionization chambers (IC), in such way that an individual IC do not mask the direct path of the beam to other the IC (PTW pin point IC – Figure 2). IC measurement values are then compared with the ones calculated by the TPS (equation (1)). For data set analysis, the mean deviation is calculated as the difference between measured (*dmeas_*i*_*) and calculated dose (*dcalc_*i*_*), normalized to the maximum beam dose (*dmax*) and averaged over *N* IC positions *i*:

(1) $$\sum_{i}^{N}\;\frac{1}{N}\frac{\left|dmeas_{i}-dcalc_{i}\right|}{dmax}\%$$

The number of points, N, included in the calculation can be equal or lower than 12, depending on the data set. The TPS provides a 3D-averaged dose gradient for each IC position. Points with a calculated gradient higher than 0.04 Gy mm^−1^ are excluded from the analysis, since they could not be measured sufficiently accurately due to the finite size of the detector sensitive volume and experimental setup uncertainties. For QA measurements in reference conditions, the applied acceptance threshold is 5% for both mean deviation and SD over a data set.

### 2.2 Monte Carlo Simulations

FLUKA is a multipurpose MC transport code originally designed for high-energy physics but with extensive use in medical applications (10, 11). For the purpose of this paper, the HADROTHErapy suite of physical settings (known as Defaults) was selected. All geometry updates and modifications were completed with the aid of FLAIR (a graphics user interface of FLUKA).

#### 2.2.1 Current MC-Based Plan Verification

**Figure 3:**
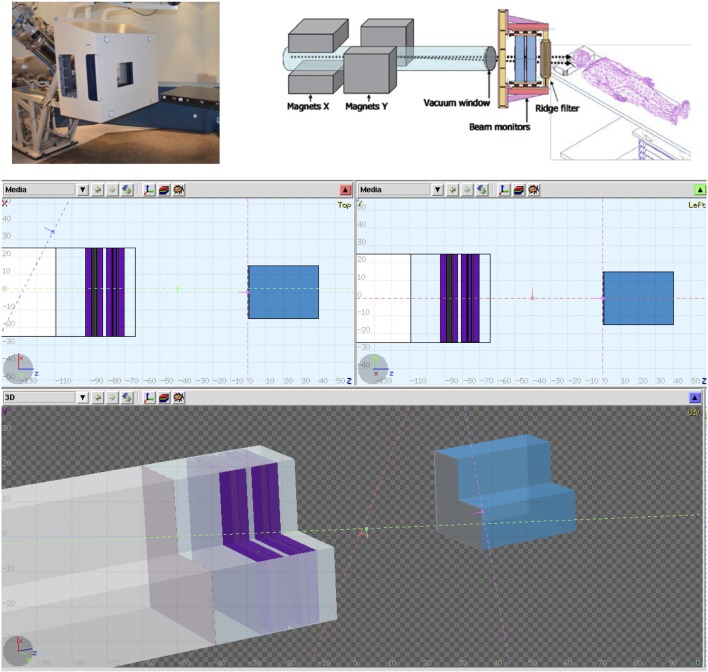
**CNAO dose delivering system can be seen in these figures**. A photo of the system, a model with its components description, and the final model used in the Monte Carlo simulations.

A complete detailed description of CNAO facility, including accelerator design and rooms layout, can be found in the literature (1, 7). For the simulation purposes, the geometry description accounted for the different structures, mainly from the monitors of the DDS, present in the beam path. The validation of this DDS description with FLUKA has been described previously (1). In Figure 3, the photo of the end of the nozzle in one of the treatment rooms is shown together with its description in a 3-dimensional model and its description within the FLUKA simulation.

Current MC patient plan verifications, as per TPS, use a simpler approach to geometrically represent the IC when calculating the dose deposition. All detectors’ structures and holders are not included, and the detector dose is sampled from the dose distribution in a water tank. By doing that, the structure and materials of the IC are not taken into consideration for the simulation. MC obtains the deposited dose in the chambers by calculating the average dose to water over several voxels, corresponding to the active volume of the detector, situated in the positions where the chambers is located.

#### 2.2.2 New Detailed Geometry

**Figure 4:**
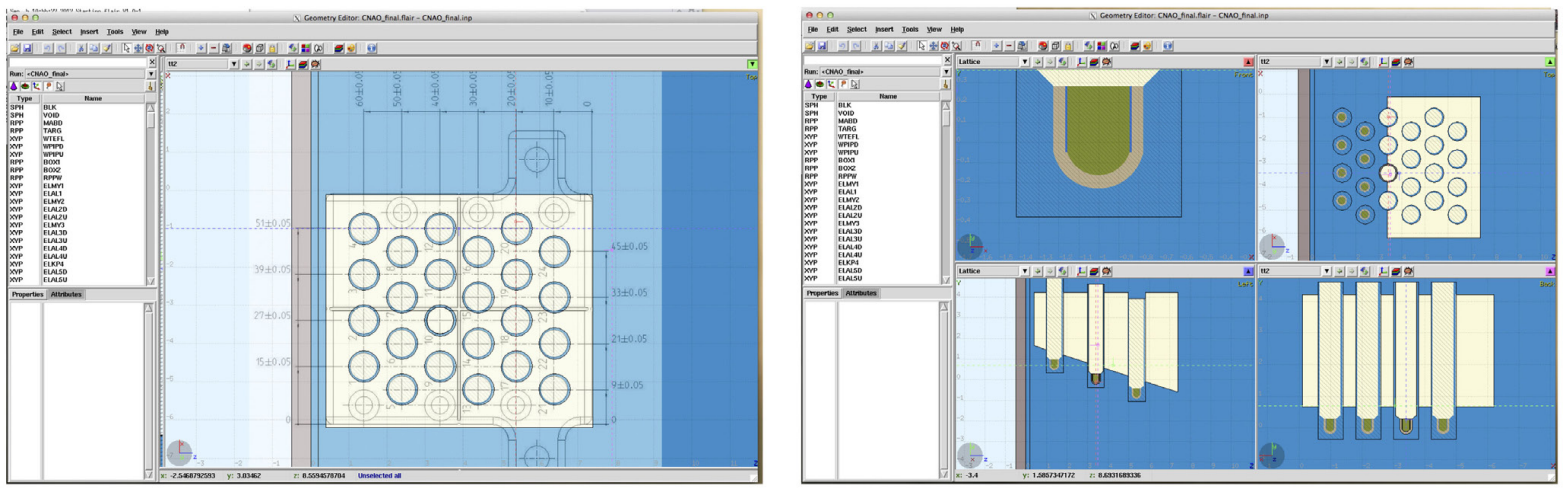
**Demonstration of the powerful tools available in FLAIR**. On the left, the technical drawing superimposed on the generated geometry is shown, and on the right, the final geometry of the phantom in 4 different views is reported.

The previously described approach, with its geometric approximations and simplifications, obtained deviations below 3% for the majority of studied cases. But for these nine cases where the agreement between the TPS calculations and measurements was above this threshold, we decided to investigate the impact of using a detailed geometry in order to account for the dose disturbances, mainly from scattered particles produced in the wall of the IC and detector holder. In the new detailed geometry, all geometry described above is kept with the inclusion of the PTW3D block and IC description (respecting all structures, dimensions, and material compositions). Detailed technical drawings were obtained from the manufactures (PTW Freiburg). Flair geometry editor was instrumental and extremely helpful in dealing with drawing and 3D visualization (Figure 4). As for the original MC approach, the absolute mean dose deviation is calculated applying equation (1).

#### 2.2.3 Delta Rays

Delta rays are defined as electrons that acquire sufficiently high kinetic energies through collisions so as to enable them to carry this energy a significant distance away from the track of the primary particle and produce their own ionization of absorber atoms (19). The FLUKA “HADROTHErapy” option uses per default delta ray production and transport cuts of 100 keV. We have chosen to vary the threshold limits in order to evaluate if the observed variation between measurements and FLUKA simulations was influenced by the delta rays threshold value.

The dose to water was calculated by averaging the dose deposited in the sensitive volume of the IC. And in order to study the effect of the delta rays threshold, all regions surrounding the sensitive volume had their threshold changed. In this work, we studied the effect of using 10 and 1000 keV in comparison to the default of 100 keV.

#### 2.2.4 Organization of This Work

**Figure 5:**
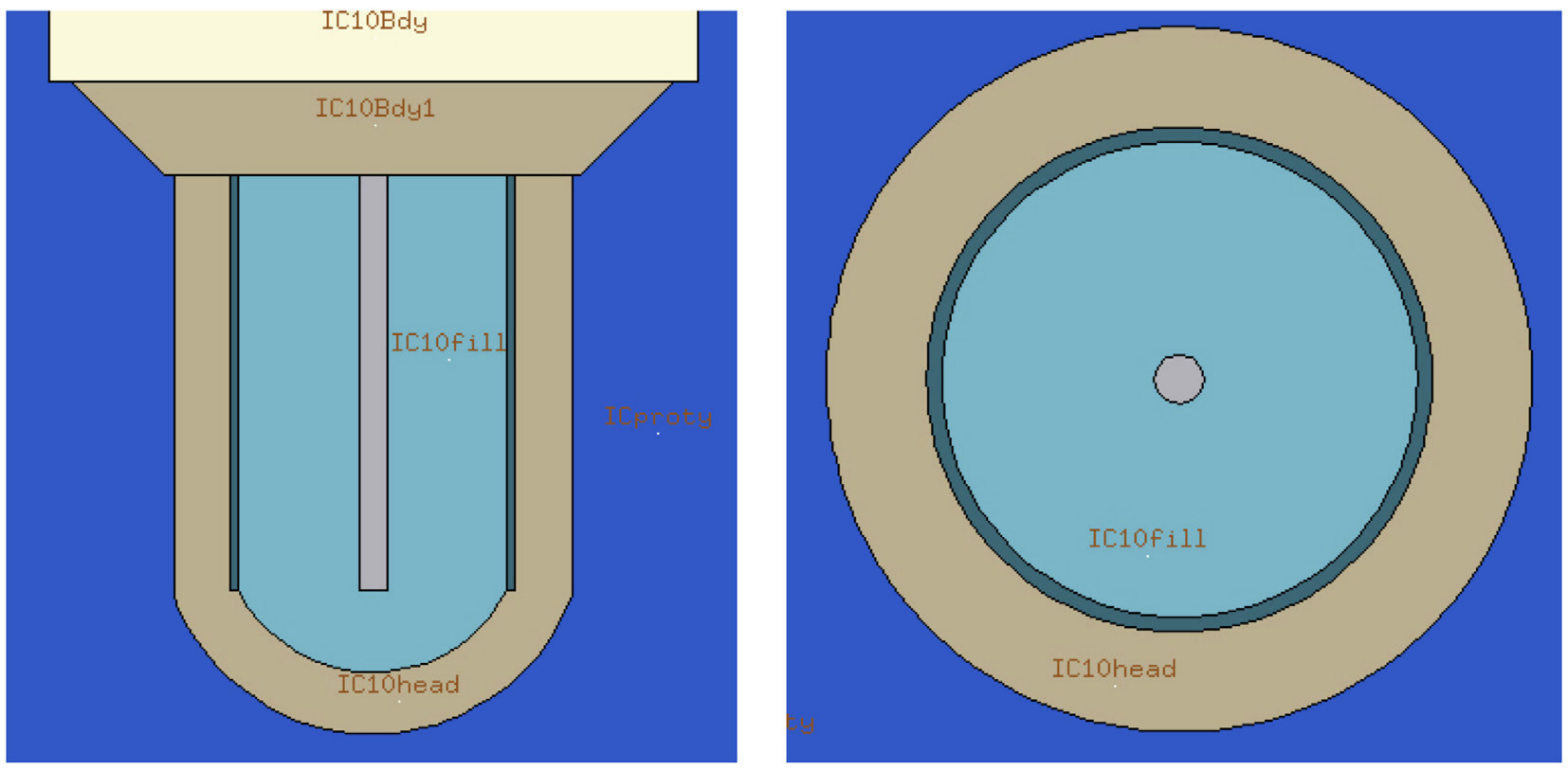
**Flair representation of internal structures of the ionization chamber**. The detector active volume is labeled as “IC10fil” for the IC number 10. All regions in, or in direct contact with, the active volume had its threshold modified for the purpose of these simulations.

In total, nine fields from different patients’ plans were analyzed, including MC simulations (for both described geometries) and dose deposition matrices from TPS and IC results (from plan verification quality assurance). The analysis of the data and this section are divided as follows:

- *Geometry effect – Section 3.1*: compared data obtained from the MC with and without the complete geometry, with experimental data and TPS calculated dose values.
- *Influence of the *δ*-rays thresholds – Section 3.2*: compared data obtained from MC simulations of two different fields for different thresholds (10, 100, and 1000 keV). For that, all regions in, or in direct contact with, the active volume had its threshold modified for the purpose of these simulations (Figure 5).
- *Chamber–chamber effect – Section 3.3*: at CNAO, the measurements are made with half of the proposed number of the detectors (24); so data from MC simulations were compared with two different setups, one with all 24 IC versus the same setup with 12 IC.

## 3 Results

### 3.1 The Influence of a Detailed Geometry Implementation

**Figure 6:**
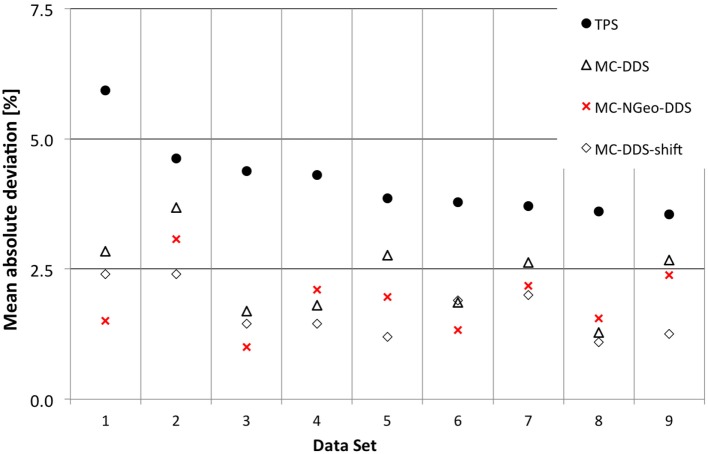
**Results for the effects of different approaches when performing patient plan verification in relation to geometry description**.

In Figure 6, it was compared for each data set results obtained implementing detailed geometry, Section 2.2.4, in relation to the ones obtained by Molinelli et al. (1).

An advantage was noticed when using a more detailed geometry (MC-NGeo-DDS), as it can be seen by the 6 cases where better results were obtained in comparison to the previous MC-DDS (MC simulations based on DDS log files). In order to understand if the difference between these MC simulations and if the measurements are significant, the relative difference between MC and measurements was calculated and analyzed.

A *p*-value of 0.003 was obtained between MC-DDS (current MC geometry description) and MC-NGeo-DDS (more detailed geometry description) by using a 2-tailed *t-test*, which describes that the obtained results by the more detailed geometry approach are significantly better in relation to the current MC Geometry description.

### 3.2 The Influence of Delta Rays Threshold

**Figure 7:**
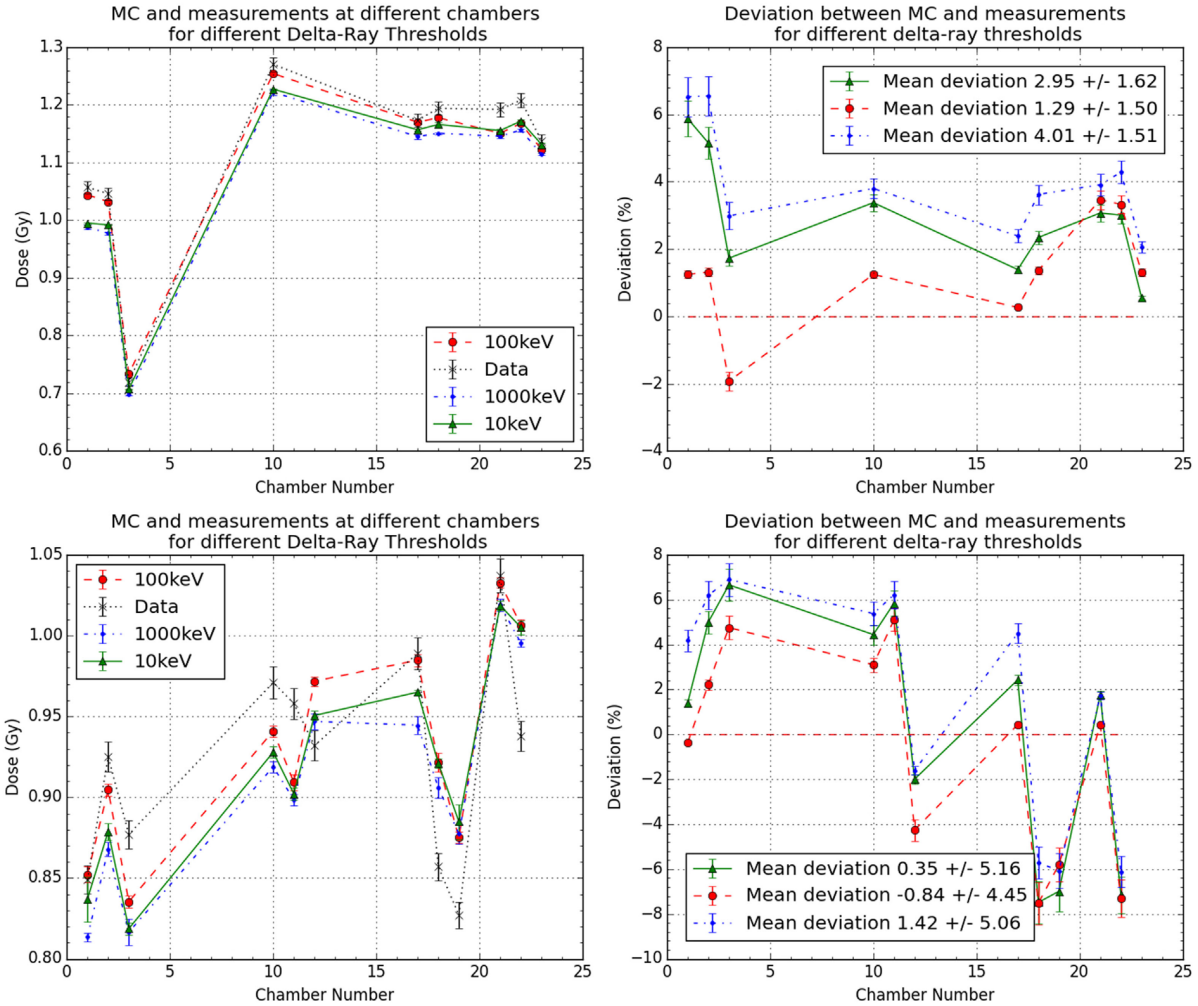
**Results for the effects of different delta rays thresholds in the more detailed geometry MC simulations for data sets 1 and 2**. It is plotted as the comparison between measurements and MC with different thresholds for the two cases (left) and their obtained deviations in respect to measurements (right).

The effect in the absorbed dose and computational time was analyzed for two patients’ data sets with different δ-ray thresholds. As described previously, in Section 2.2, with FLUKA MC code, the user is able to set different thresholds for both production and transport of different particles. Initially as expected, some differences were noted between the individual measurements for each threshold and comparison to the current default threshold, set at 100 keV (Figure 7). When compared to the measurements individually and as data set (Figure 7), no comparable advantage was noticed by using different thresholds.

When comparing the computational time when the δ-ray threshold is changed, it was observed that by increasing the threshold (from 100 to 1000 keV), the average time to simulate all primaries reduced by 11.45 ± 3.39%, and when the threshold was reduced (from 100 to 10 keV), the average time to simulate all primaries increased by 43.49 ± 22.02%.

### 3.3 Chamber–Chamber Effect

**Figure 8:**
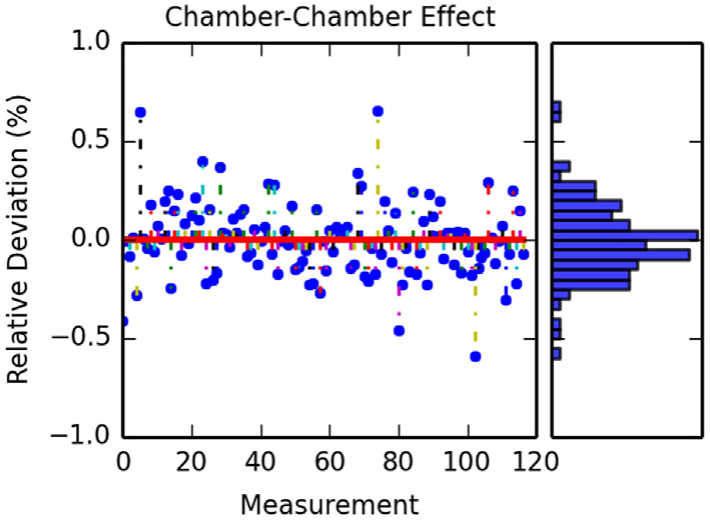
**Calculated differences between simulation with 12 and 24 ionization chambers**. The figure on the left shows the different deviation for each IC measurement for all the data sets. The figure on the right shows the obtained histogram of these deviations.

Another aspect analyzed was the fact that instead of using the full 24 positions available in the ionization chambers holder (see Figure 4), only 12 positions were used, allowing for investigating the influence of chamber–chamber effect. A 2-tailed *t-test* was performed, as in Section 3.1, and no statistical significant difference (p-value 0.996) was found between both simulations with 12 or 24 chambers. Figure 8 shows the calculated deviations for the different data sets for both MC and TPS.

## 4 Discussion

### 4.1 The Influence of a Detailed Geometry

In this work, we evaluated the effect of the IC geometry description in MC simulation for patient plan verification. We compared our geometrical description with the current approach used. Figure 6 showed that by improving the details of the detectors geometry description, on average, we obtained a mean deviation of 1.90% with 0.63% 1 SD for the 9 cases in comparison to the current method (1), which obtained a mean deviation of 2.36% (0.75%).

Another source of uncertainty is the positioning of the phantom. In order to evaluate if the deviations found were introduced not only by the MC simulations but also by the position of these detectors during measurements, we simulate the effect of introducing this uncertainty.

We simulated different detector positions within ±1 mm in all direction for one of the data set (Data Set 2) around the original position, in total, 27 positions miming uncertainties in the detector positioning. A new minimum of 1.94% was found at (1, −1, 1) as dx, dy, and dz, respectively, in comparison to 2.40% as reported by Molinelli et al. (1).

**Figure 9:**
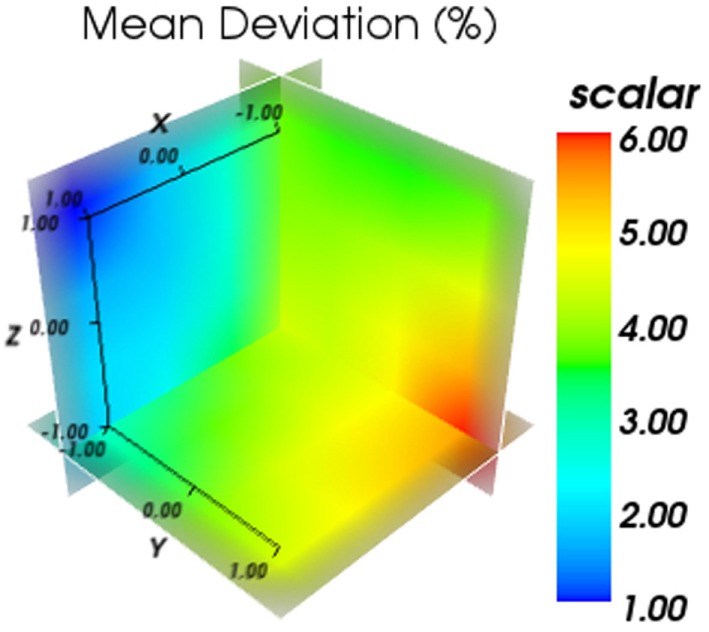
**Results for the obtained mean deviation in respect to the applied offset**. The color bar represents the mean average deviation obtained in %.

The obtained mean deviation in respect to the applied offset can be seen in Figure 9. It can be seen that the obtained deviation varies with the positioning of the detector block in a systematic manner, where a minimum and a maximum deviation can be obtained by optimizing the detector position. This also shows that for this data set, the importance of a proper positioning of the water phantom.

### 4.2 Chamber–Chamber Effect

In addition to the benefits of using a more detailed geometry description, additional points needed to be evaluated as possible contribution to errors and uncertainties. The first one was the interference seen by a detector from the interaction of beam to previously positioned detectors. Although this effect had been evaluated for carbon ions (20) in the case of protons which are more susceptible to the broadening, it had not been evaluated. In our study, no significant difference was seen between the measurements with and without the extra detectors.

### 4.3 The Influence of Delta Rays Threshold

Another possible factor which will influence the MC simulation results with more detailed detector geometry is the choice of delta rays threshold. For this reason, we evaluated threshold in our detailed geometry. We found that current thresholds used by the default, which have been previously analyzed (11), are still sufficient for this detailed geometry, and no improvement was observed by the reduction of these.

## 5 Conclusion

The use of MC simulations in aiding patient plan verification has been evaluated. In this work, we studied the effect of improving the detectors geometry description in the MC simulations. We showed that even in the most challenging scenarios of very non-uniform fields, a more detailed geometric description of the detectors results in better agreement with the measurements, although at the cost of more computational time (18.8% in average). If taken into considerations that only 9 patient in an entire year period crossed the threshold, this increase of time should not limit the use of a more detailed geometry description. Additionally, we saw that for few cases where the uncertainty of mispositioning was more relevant than the modeling uncertainties, the use of detailed geometry description in the MC simulation was not able to improve agreement with measurements. For these cases, it was only possible to obtain a better agreement after the detector position was shifted.

## Author Contributions

All the authors participated in the different stages of the work, from conception, analysis, and writing.

## Conflict of Interest Statement

The authors declare that the research was conducted in the absence of any commercial or financial relationships that could be construed as a potential conflict of interest.
